# Extending the OMOP Common Data Model and Standardized Vocabularies to Support Observational Cancer Research

**DOI:** 10.1200/CCI.20.00079

**Published:** 2021-01-07

**Authors:** Rimma Belenkaya, Michael J. Gurley, Asieh Golozar, Dmitry Dymshyts, Robert T. Miller, Andrew E. Williams, Shilpa Ratwani, Anastasios Siapos, Vladislav Korsik, Jeremy Warner, W. Scott Campbell, Donna Rivera, Tatiana Banokina, Elizaveta Modina, Shantha Bethusamy, Henry Morgan Stewart, Meera Patel, Ruijun Chen, Thomas Falconer, Rae Woong Park, Seng Chan You, Hokyun Jeon, Soe Jeong Shin, Christian Reich

**Affiliations:** ^1^ Memorial Sloan Kettering, New York City, NY; ^2^ Clinical and Translational Sciences Institute, Northwestern University, Evanston, IL; ^3^ Regeneron Pharmaceuticals, Tarrytown, NY; ^4^ Odysseus Data Services, Cambridge, MA; ^5^ Tufts Clinical and Translational Science Institute, Boston, MA; ^6^ Tufts Institute for Clinical Research and Health Policy Studies, Boston, MA; ^7^ Real World Solutions, IQVIA, London, United Kingdom; ^8^ Vanderbilt University Medical Center, Nashville, TN; ^9^ University of Nebraska Medical Center, Omaha, NE; ^10^ National Cancer Institute, Bethesda, MD; ^11^ Department of Biomedical Informatics, Columbia University, New York City, NY; ^12^ Department of Biomedical Informatics, Ajou University School of Medicine, Suwon, South Korea

## BACKGROUND

The Observational Health Data Sciences and Informatics (OHDSI) community conducts research on a global scale using standardized representations of healthcare data and reproducible standardized open source analytics.^1^

**CONTEXT**

**Key Objective**
To develop an extension of the OMOP Common Data Model and Standardized Vocabularies to support the comprehensive representation of cancer conditions, treatments, and disease abstractions required for addressing key research questions.
**Knowledge Generated**
Developed and tested the OMOP Oncology Extension that supports granular representation of cancer diagnoses and treatments and clinically relevant disease and treatment episodes and outcomes. Integrated terminologies that provide comprehensive coverage of the oncology domain into the OMOP Standardized Vocabularies. Developed vocabulary-driven transformation from US Tumor Registries into the OMOP Common Data Model.
**Relevance**
The OMOP Oncology Module provides a platform for standardization of cancer data enabling the conduct of observational cancer studies and identifying patient cohorts in a distributed research network. Incorporated vocabularies create a foundation for manual or automated abstraction of cancer data to identify larger disease episodes and outcomes and enable automated transformation of the source data.

Systematic, standardized analytics requires harmonization of the data, which also enables distributed network research.^2^ Both the data model (structure) and the concept representation (terminology) need to be standardized to support federated analytics. Data standardization methods and tools are an integral part of the OHDSI research infrastructure. In the heart of it is the OMOP Common Data Model (CDM) and Standardized Vocabularies,^3^ demonstrably the most widely used CDM around the world.^4^ Each institution participating in OHDSI converts its data into the OMOP CDM and Standardized Vocabularies, retaining the data in-house, thus enabling federated conduct of queries, hypothesis generation, and observational studies without having to share sensitive patient-level data with other institutions.

In a typical observational study, cohorts, exposures, and outcomes can be sufficiently well defined through the presence or absence of clinical events encoded by a defined set of concepts.^3,5^ Observational research in cancer is more challenging than that of most other conditions for a number of reasons^6^:

1. Cancer diagnoses are explicitly defined through a set of attributes including histology, anatomic site of origin (topology), extent of disease spread (stage), degree to which the cancer microscopically resembles its tissue of origin (grade), and cancer-specific biomarkers. These constellations of attributes determine patients’ prognosis and treatment options.
2. Cancer treatments are often administered in chemotherapy regimens with complex dosing and scheduling in multiple cycles and are often combined with targeted therapies, immunotherapies, surgery, or radiotherapy. Frequently, regimens are personalized to the individual patient’s needs, making the process of standardizing them more complex.
3. Patient’s trajectory through the disease is organized into episodes of diagnosis, treatment, and outcome with disease-free survival, relapse or recurrence, and progression.^7^ Estimates and prediction of cancer survival, the ultimate outcome, rely on the availability of the death data, notoriously under-reported in observational data.

Currently, the OMOP CDM does not adequately cover cancer diagnoses, treatments, and episodes. There is also no comprehensive cancer data model or terminology available in the public domain that would support harmonization of cancer data to enable standardized analytics in a research network. In this paper, we introduce the OMOP Oncology Module that extends the OMOP CDM and Standardized Vocabularies to support the comprehensive representation of cancer conditions, treatments, and disease abstraction required for addressing key research questions.

## METHODS

To represent an adequate cancer model, we had to extend the OMOP CDM. In particular, the logical data model and Standardized Vocabularies had to be extended to represent each of the three key components: cancer diagnoses, cancer treatments, and cancer episodes. Extensions of the data model had to be backward-compatible in such a way that existing OMOP data instances would not be affected while the utility of the extension would not be limited. Extension of the Standardized Vocabularies required integration of terminologies to cover the new semantic space under the established OMOP ontological design principles. In particular, concepts must be (1) precise and sufficiently granular, (2) unambiguous and nonredundant, (3) comprehensive in their coverage of the semantic space, and (4) represented in an ontology, which can be integrated into the existing ontologies.^8^

We considered concepts from seven existing standards:

- WHO International Classification of Diseases for Oncology, 3rd Edition (ICD-O-3),^9^ international standard classification of tumor histology, topology, and behavior used for reporting to tumor registries.
- HemOnc.org—A Free Hematology/Oncology (HemOnc),^10^ ontology of cancer therapy regimens.
- North American Association of Central Cancer Registries (NAACCR),^11^ consolidated data dictionary from Data Standard Setters SEER, Commission on Cancer, Centers for Disease Control's National Program of Cancer Registries, and Cancer Center–based Clinical Registries (CCCR) for cancer registration and surveillance, containing site-specific data items required for staging or the standard setting agencies.
- College of American Pathologists (CAP) electronic Cancer Checklists,^12^ systematic checklist for collecting essential data elements for pathology reporting and staging of malignant tumors.
- Nebraska Medicine Clinical Ontology Application (Nebraska Lexicon),^13^ extension of SNOMED Clinical Terms (CT) to cover concepts necessary for pathology synoptic reporting.
- National Cancer Institute Thesaurus (NCIt),^14^ reference terminology for NCI systems, containing terminologies for clinical care, translational and basic research, public information, and administrative activities.
- Anatomical Therapeutic Chemical^15^ Drug classification system based on the affected organ or system and therapeutic, pharmacological, and chemical properties.

### Cancer Diagnosis Model

**FIG 1.**
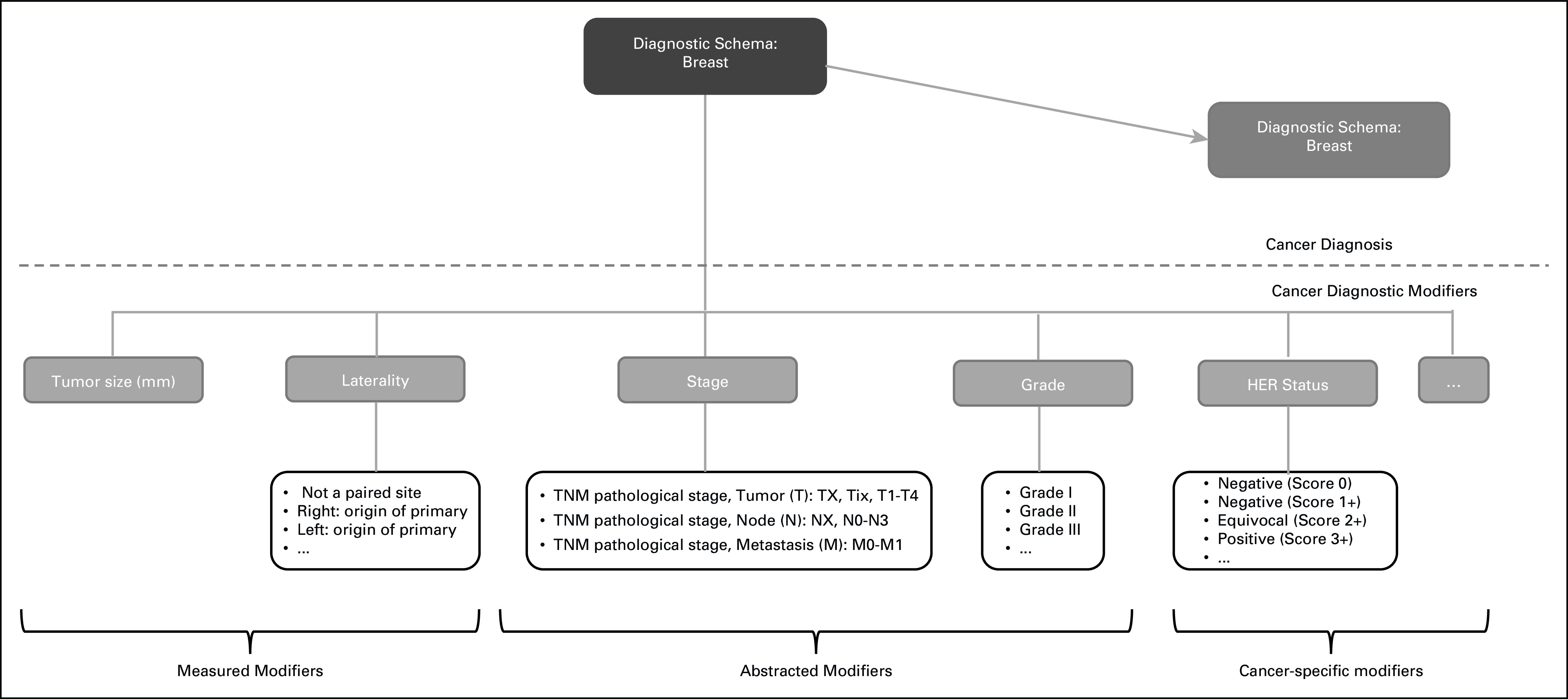
Cancer diagnosis model. HER, human epidermal growth factor receptor.

We grouped cancer diagnoses into broader models each based on cancer type defined by their macroscopic (topology) or microscopic (histology) origin (eg, breast cancer and lymphoma). Each cancer type covers a set of diagnoses that share the same attributes or diagnostic modifiers (Fig 1). Each cancer diagnosis is uniquely defined by its topology and histology. Cancer modifiers can be measured (eg, tumor size), abstracted (eg, tumor stage), and cancer-specific (eg, human epidermal growth factor receptor 2 for breast cancer).

#### Logical data model for cancer diagnoses

**FIG 2.**
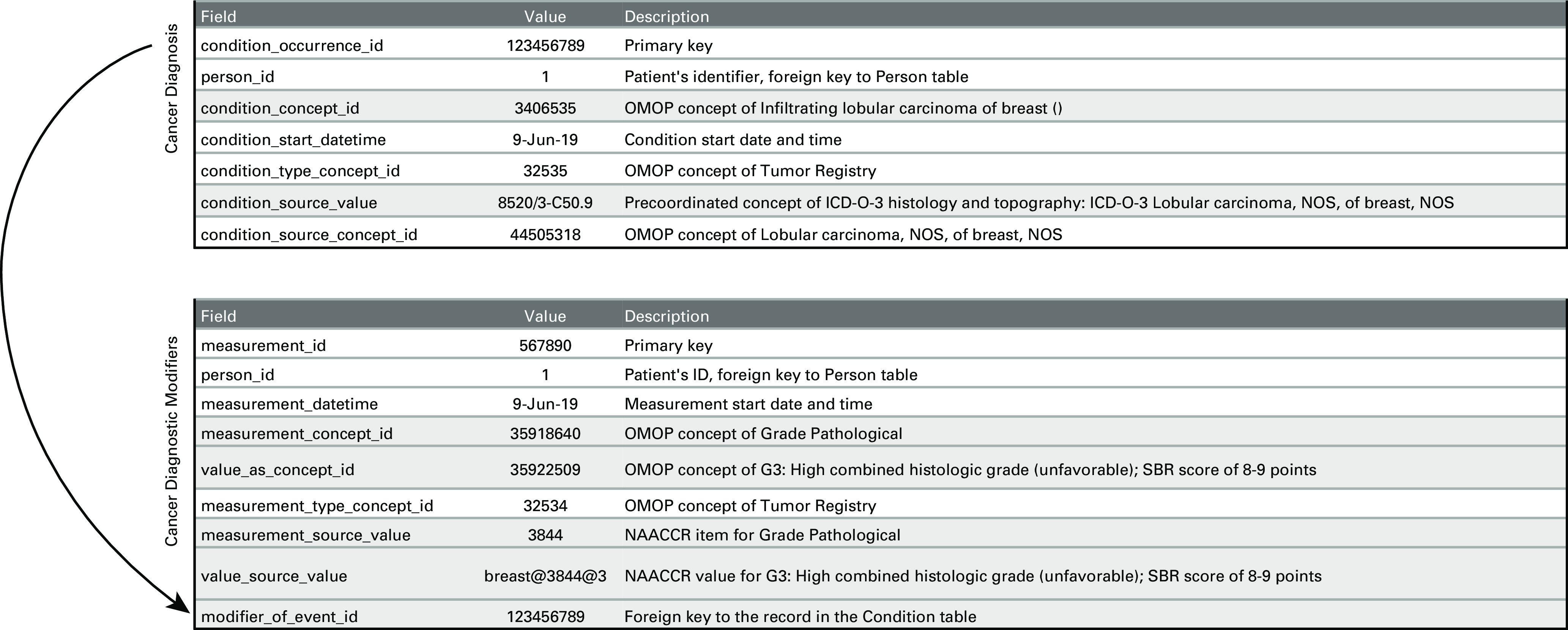
Linkage between cancer diagnosis and diagnostic modifiers in OMOP Common Data Model. ICD-O-3, International Classification of Diseases for Oncology 3rd Edition; NAACCR, North American Association of Central Cancer Registries; NOS, not otherwise specified; SBR, Scarff-Bloom-Richardson.

We chose to represent cancer diagnoses as conditions in the CONDITION_OCCURRENCE table in the data model and cancer modifiers as measurements in the MEASUREMENT table in the data model. The consequence of this choice was that diagnoses must be fully precoordinated concepts containing all detail of topology and histology, while cancer modifiers could also be postcoordinated into concepts and values. The only addition to the MEASUREMENT table was an explicit link to the cancer diagnosis in the CONDITION_OCCURRENCE table (Fig 2).

#### Standard concepts for cancer diagnoses

Currently, most standard concepts of the Condition domain in the OMOP Standardized Vocabularies are sourced from the SNOMED CT disease ontology. Diagnoses coded in other terminologies (eg, ICD-9/10) in the source data, including cancer diagnoses, are mapped to SNOMED CT during conversion to OMOP. SNOMED CT is a rich disease ontology that provides comprehensive representation of noncancer diagnoses and supports a wide range of analytical use cases. However, SNOMED CT is not granular enough to cover all the topology and histology details.^13^

**FIG 3.**
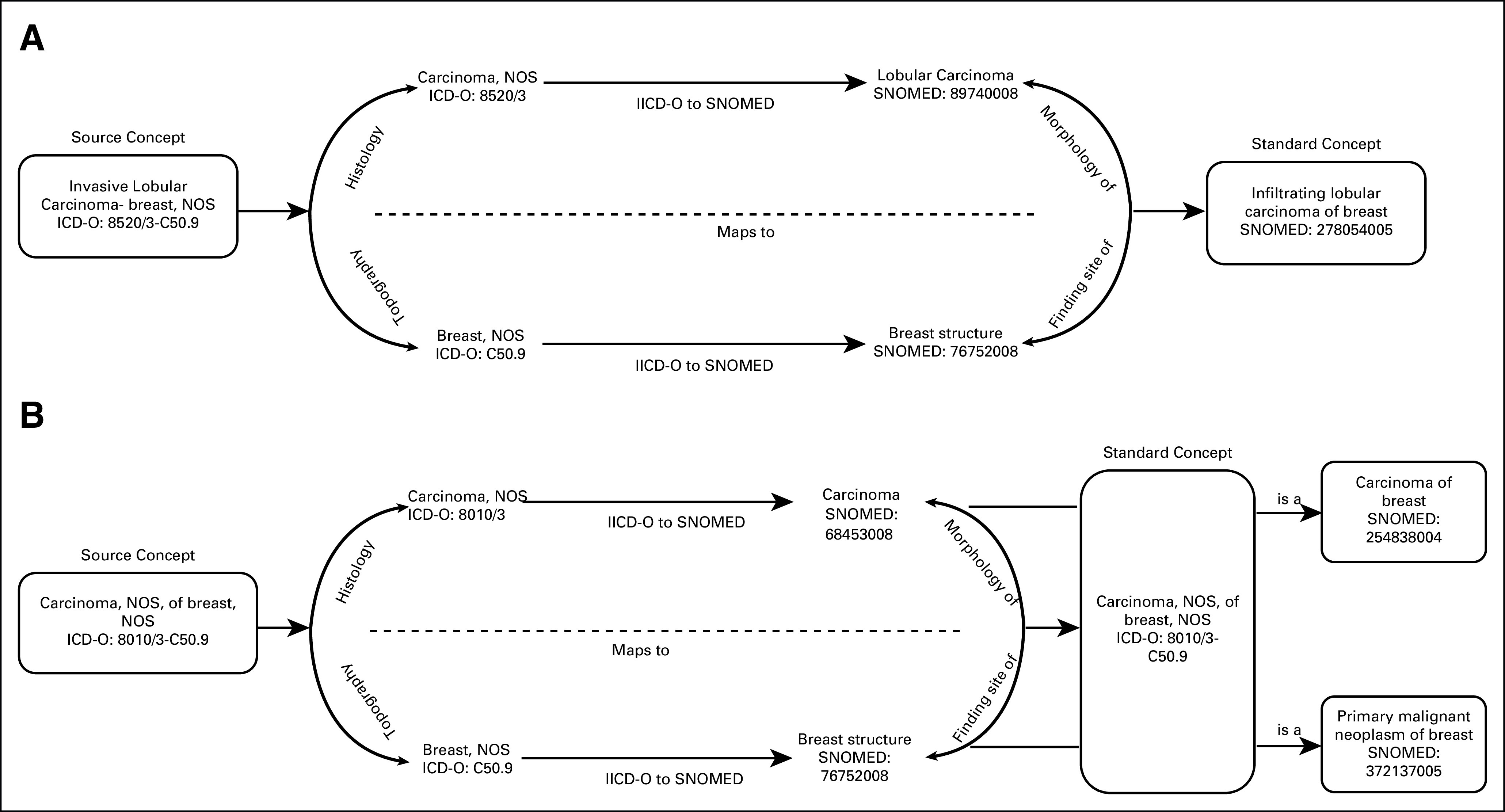
Mapping precoordinated International Classification of Diseases for Oncology (ICD-O), 3rd Edition, histology and topography concepts to SNOMED CT: a) exact match found and b) exact match not found. NOS, not otherwise specified.

WHO ICD-O-3 is the international standard used in reporting to tumor registries.^9^ It provides specialized representation of cancer histology, topography, and behavior obtained from pathology reports. We chose ICD-O-3 to represent detailed cancer diagnoses and precoordinated ICD-O-3 histology, topography, and behavior into single condition concepts. Of all the theoretically possible combinations, we only instantiated those that were observed in the SEER-reported combinations of these dimensions.^16^ In addition, we integrated these precoordinated ICD-O-3 combination concepts into the SNOMED CT hierarchy. Our integration approach was two-step. First, we established equivalence between the ICD-O-3 *histology* and *topography* and the respective SNOMED CT–*associated morphology* and *finding site* attributes. Then, we matched the precoordinated ICD-O-3 concept with the respective SNOMED CT disorder at the intersection of equivalent *associated morphology* and *finding site* attributes. If exact equivalence between the two concepts was established, the mapping between the ICD-O-3 and SNOMED CT concepts was integrated in the vocabulary and the SNOMED CT concept would become a designated standard concept for this diagnosis. For example, ICD-O–based concept 8520/3-C50.9 (lobular carcinoma, NOS [not otherwise specified], of breast, NOS) is an exact equivalent of SNOMED CT concept 278054005 (infiltrating lobular carcinoma of breast). Therefore, it was mapped directly to the SNOMED CT concept (Fig 3A). If exact equivalence between the two concepts was not established, the precoordinated ICD-O-3 concept was integrated into the SNOMED CT disease ontology as a disorder concept at the intersection of the *associated morphology* and *finding site* attributes identified in the first step and was placed as a child of the existing SNOMED CT disorder at the intersection of the two axes. For example, the ICD-O–based concept 8010/3-C50.9 (carcinoma, NOS, of breast, NOS) was integrated into the SNOMED CT ontology at the intersection of the *finding site* concept 76752008 (breast structure) and *associated morphology* concept 68453008 (carcinoma) and placed in the hierarchy as a child of the SNOMED CT concept 254838004 (carcinoma of breast) (Fig 3B). This approach enriched the existing SNOMED CT concept space with much more detailed cancer diagnoses, but still preserved the disease hierarchy, making existing queries backward compatible with the new cancer model.

Finally, we linked precoordinated diagnosis concepts to the respective cancer type. For example, precoordinated ICD-O-3 *carcinoma, NOS, of breast, NOS (8010/3-C50.9)* was linked to the concept of cancer type: breast.

#### Standard concepts for diagnostic modifiers

Our evaluation of American Joint Committee on Cancer, CAP, NAACCR, and NCIt for the representation of diagnostic modifiers revealed that there is no single terminology that provides complete coverage and is fit for our purpose. Instead, we adopted the standard authoring process of Nebraska Lexicon,^13^ in which we mapped nonontological standards such as CAP and NAACCR to one ontological representation.

### Cancer Treatment Model

In the OMOP CDM, treatments are represented through the Procedure and Drug domains, allowing to record individual surgery, drug exposure, or radiotherapy events. While this is sufficient for other therapeutic areas, therapy regimens are required for representation of cancer treatments. We integrated HemOnc ontology as a reference for regimens into the OMOP Standardized Vocabularies and mapped regimen components to standard RxNorm^17^ concepts.^10^

### Cancer Episode Model

The patient’s journey through the disease includes a typical sequence of disease states and treatments, which are foundational end points for cancer research. We called these episodes. Episodes may be composed of multiple individual clinical events. We introduced a new logical model and respective concepts into the OMOP CDM to represent this domain.

#### Logical model for cancer episodes

**FIG 4.**
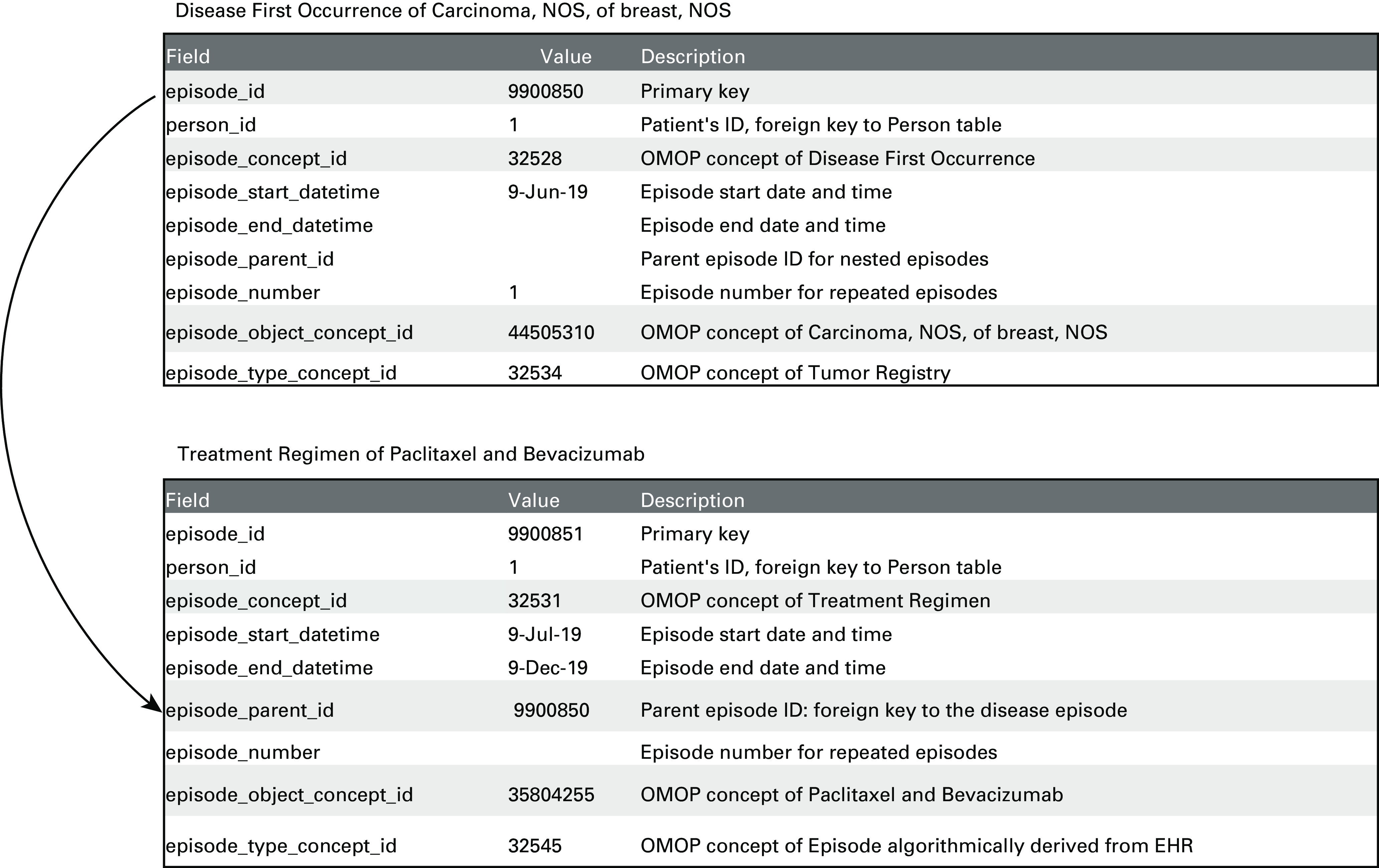
Disease and treatment episodes in OMOP Data Model. NOS, not otherwise specified.

We created new EPISODE and EPISODE_EVENT tables. Each episode record contains a concept of abstracted disease state (eg, disease first occurrence and treatment regimen) along with a concept of a specific cancer diagnosis (eg, carcinoma, NOS, of breast, NOS) or treatment (eg, paclitaxel and bevacizumab) (Fig 4). Episodes can be nested. For example, a cancer disease episode might include several disease states (eg, stable disease and disease progression), and a treatment regimen episode may include multiple regimen cycles. If known, individual clinical events of the Condition, Drug, and Procedure domains may be linked to episodes they comprise through the EPISODE_EVENT table.

#### Standard concepts for cancer episodes

We generated de novo concepts representing disease and treatment states in the Episode domain.

#### Abstraction of episodes from clinical events

Disease and treatment episodes are immensely valuable for research but are rarely available in a structured form in the majority of data sources, with some exceptions in tumor registries. Rule-based and probabilistic methods of cancer episode derivation are evolving.^18-20^ We developed and deployed a rule-based algorithm that matches drug ingredients, their timing, and dosing from individual medication records to the appropriate treatment regimens defined in HemOnc.

### Conversion From Tumor Registry to OMOP CDM

We developed a vocabulary-driven extract-transform-load (ETL) process for converting US Tumor Registry data into OMOP CDM.^21^ NAACCR data dictionary integrated into the Standardized Vocabularies served as a basis for this ETL.

### Use Case: Characterization of Bladder Cancer

We assessed the utility of the OMOP Oncology Module to generate real-world evidence (RWE) as a part of an ongoing observational study. Data from electronic health records (EHRs) and cancer registries from Ajou University, IQVIA Oncology Electronic Medical Record, and IQVIA OpenClaims were used to identify patients with metastatic bladder cancer (mBC) treated with a systemic chemotherapy regimen and describe regimens received in the first-line therapy. The observation period extended from bladder cancer diagnosis to the date of last encounter in the database or June 25, 2020, whichever occurred first. Chemotherapy regimens were determined using the chemotherapy regimen detection algorithm.

## RESULTS

We evaluated seven vocabularies for their suitability to serve as standard concepts for the cancer model. Each terminology was assessed for the presence of concepts in each category (Presence), comprehensive coverage of the category (Completeness), and adherence to the ontological principles using the Nebraska Lexicon mapping approach (Table 1).

**TABLE 1.**
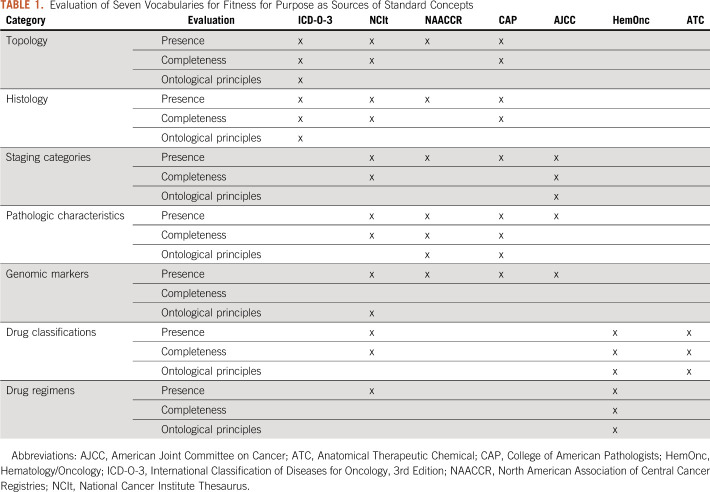
Evaluation of Seven Vocabularies for Fitness for Purpose as Sources of Standard Concepts

No single terminology alone covers the entire semantic space with the exception of the NCIt. No single terminology, with the exceptions of ICD-O-3 and HemOnc, provides fit for purpose coverage and adheres to the ontological principles out of the box. As a result, as of today, we implemented ICD-O-3 as standard concepts for Cancer Diagnoses and HemOnc as standard concepts for regimens. We created an initial set of concepts for breast and prostate cancer diagnostic modifiers using and extending the Nebraska Lexicon.

We tested the data conversion at six participating institutions including Memorial Sloan Kettering, Northwestern University, Tufts Clinical and Translational Science Institute, IQVIA, Columbia University Medical Center, and Ajou University School of Medicine. All participating sites successfully converted their raw data into the OMOP Oncology representation, combining Tumor Registry and EHR data, and passed quality assurance testing. All analytics were generated centrally, distributed to the institutions, and executed locally. Completeness of Tumor Registry diagnosis coverage using ICD-O-3 precoordinated concepts ranged from 96% to 100% at four participating institutions: Northwestern University, Tufts University Medical Center, Columbia University Medical Center, and Memorial Sloan Kettering.

Integration of Tumor Registry data with the EHR data allowed for combining granular clinical data with important metrics not routinely available in EHR including tumor stage and pathology. This linkage also improved data completeness. Specifically, incorporation of information on patients’ vital status provided basis for a more accurate survival estimation. At Northwestern University, this integration led to the identification of 75 additional deaths, which is a 26% increase in death count in the linked data. At Memorial Sloan Kettering, linkage between Tumor Registry data and EHR resulted in 2,655 additional deaths, which is a 4% increase in death count.

**FIG 5.**
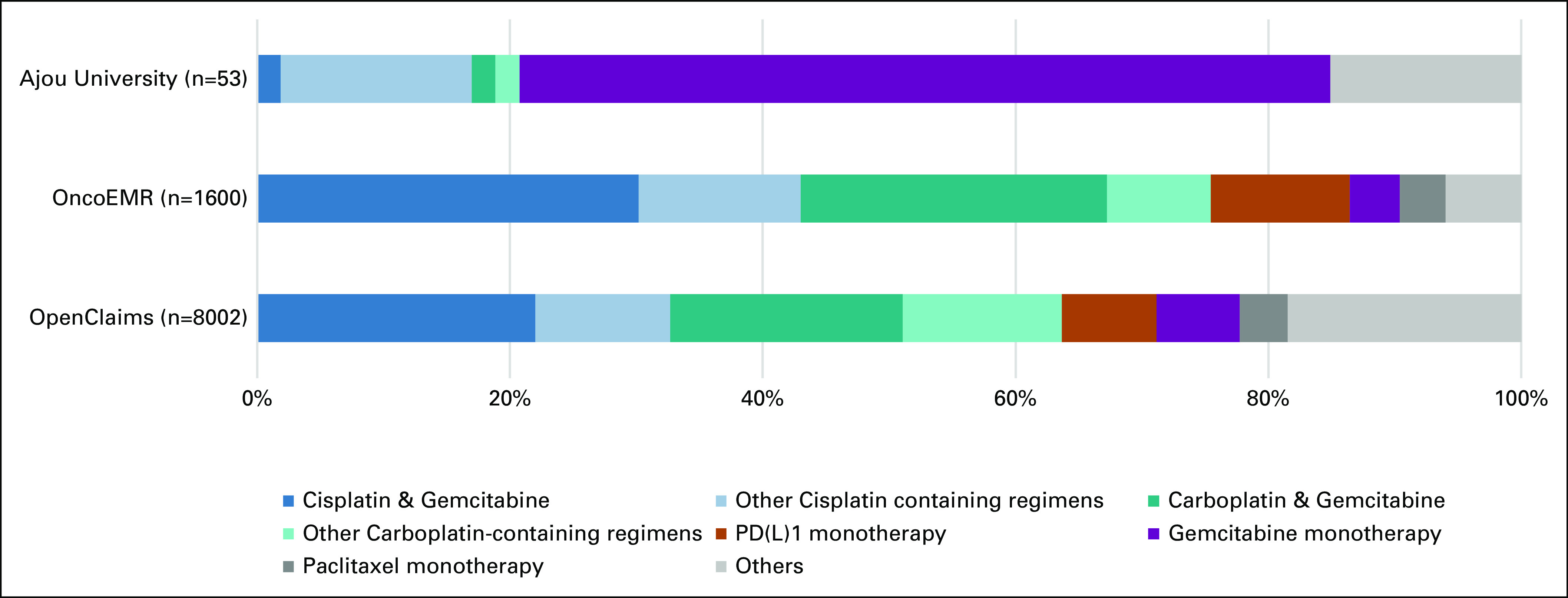
Distribution of treatment regimens in the first-line therapy for patients with metastatic bladder cancer in three select network participants. The various regimens were combined into six treatment categories according to the National Comprehensive Cancer Network recommendation.^22^

Patients with mBC were identified by a standardized cohort definition in participating centers. Median age at the time of first encounter with mBC ranged from 65 to 71 years, and 74% to 81% of patients were male. Median duration of the first-line therapy was between 42 and 55 days across the centers. Distribution of treatment regimens in the first-line therapy for patients with mBC is presented in Figure 5.

## DISCUSSION

In the OMOP Oncology Module, we achieved the goal of representing cancer diagnoses and treatments at a granularity required for conducting observational cancer studies and identifying patient cohorts.

The OMOP Oncology Module episode model enables representation and analysis of clinically relevant disease and treatment episodes and outcomes. It also equips researchers with the foundation to test, compare, and validate various algorithms of episode derivation.

Integration of the NAACCR data dictionary into OMOP and development of a standardized vocabulary-driven ETL-enabled OHDSI network with the ability to bring US Tumor Registry data into OMOP CDM in a uniform automated fashion.

Incorporation of the HemOnc ontology into the Standardized Vocabularies enabled the development of algorithms for derivation of systemic treatment regimens.

Adoption and testing of the OMOP Oncology Module at six participating institutions demonstrated successful data conversion. Combining EHR with Tumor Registry data improved data granularity and completeness and enabled the identification of the first cancer occurrence and the first-line treatment not easily identifiable in the EHR data alone.

The overall feasibility of the OMOP Oncology Module to generate RWE in a distributed network study was successfully tested as a part of an ongoing characterization study of treatment patterns and clinical outcomes of patients with mBC. We described the real-world treatment of patients with mBC in the first-line therapy. The difference observed in the treatment between centers can be due to the existing difference in medical practice and standard of care across the collaborating centers and will be fully explored in the ongoing study. The ongoing study will incorporate data from other data partners that are in the process of improving the accuracy of the integration of their Tumor Registry data into OMOP.

However, addressing a variety of use cases (eg, precision oncology) and providing adequate foundation for comprehensive systemic analytics is still limited and requires finalizing the work in the terminology space.

One of the limitations of the described effort was the lack of a formalized ontology authoring method for integration of ICD-O-3 diagnoses into SNOMED CT and assessment of the impact of the method used on the accuracy and precision of the data converted into these vocabularies. To address this, we will apply the Nebraska Lexicon standardized authoring process in which the new concepts are defined in full detail using the current SNOMED CT concept model and then placed in the proper, logical place in the SNOMED CT polyhierarchy by the OWL classifier.

Our immediate terminology development efforts are focusing on extending terminology coverage to the domains critical for cancer, specifically genomics and radiology, and harmonizing heterogeneous representations of diagnostic attributes (CAP, NAACCR, American Joint Committee on Cancer, and NCIt) to one ontological standard.

Our next major milestone is adaption and development of the new algorithms for derivation of disease and treatment episodes and outcomes.

## Michael J. Gurley

**Stock and Other Ownership Interests:** Textractor Technologies

**Consulting or Advisory Role:** Textractor Technologies

**Patents, Royalties, Other Intellectual Property:** Developed a Case Finding tool for tumor registry operations.

## Asieh Golozar

**Employment:** AstraZeneca

**Stock and Other Ownership Interests:** Regeneron

## Jeremy Warner

**Stock and Other Ownership Interests:** HemOnc.org

**Consulting or Advisory Role:** Westat, IBM

**Travel, Accommodations, Expenses:** IBM

## W. Scott Campbell

**Travel, Accommodations, Expenses:** GenomOncology

No other potential conflicts of interest were reported.
